# Preliminary phytochemical screening and antibacterial effects of root bark of *Ferula communis* (Apiaceae)

**DOI:** 10.1002/vms3.1170

**Published:** 2023-07-01

**Authors:** Betelihem Yirdaw, Temesgen Kassa

**Affiliations:** ^1^ Assosa Agricultural Research Centre Ethiopian Institute of Agricultural Research Assosa Ethiopia; ^2^ Holeta Agricultural Research Centre Ethiopian Institute of Agricultural Research Holeta Ethiopia

**Keywords:** antimicrobial effects, inhibition zone, phytochemical, plant

## Abstract

**Introduction:**

Plants are widely used in traditional medicine because they contain a high concentration of antimicrobial agents, serving as the foundation for medicines. The aim of this study was preliminary identification of phytochemicals and assesses the antimicrobial activity of extracts of *Ferula communis* root bark.

**Methods:**

Plant was collected, and standard qualitative procedures were conducted. The plant samples were extracted with 99.9% methanol and 80% ethanol. To identify phytochemicals found in plants, a preliminary phytochemical analysis was performed. Agar diffusion tests, minimum inhibitory concentrations (MICs) and minimum bactericidal concentrations (MBCs) were performed to evaluate antibacterial activity.

**Result:**

The preliminary phytochemical analysis of the ethanol and methanol extract revealed positive results for flavonoids, coumarins and tannins. Terpenoids and anthraquinones were detected only in the methanol extract. The extract of *Ferula communis* showed an antibacterial effect on both gram‐negative and gram‐positive bacteria in a concentration‐dependent manner. The average zone of inhibition for gram‐positive bacteria was 11 mm, whereas for gram‐negative bacteria, it was 9 mm. The MIC and MBC values also varied with the type of bacteria. In all bacterial species tested, the mean MBC value was similar to the MIC.

**Conclusion:**

Different phytochemicals were detected in extracts of the root bark of *F. communis* and extracts showed antibacterial effects in a concentration‐dependent manner. Therefore, further purification and evaluation of the extracts and antioxidant activity of the plant should be investigated.

## Graphical abstract

Ethanol extracts of root bark of *Ferula communis* (Apiaceae) were positive for flavonoids, tannins and coumarins, whereas the methanol extract was positive for the presence of tannins, flavonoids, coumarins and terpenoids. The extract of *F. communis* showed an antibacterial effect on both gram‐negative and gram‐positive bacteria in a concentration‐dependent manner.

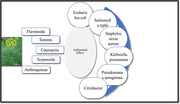

## 1 INTRODUCTION

Ethiopia has one of the largest livestock populations in Africa. Despite this, it has one of the world's lowest unit outputs. This is primarily due to livestock diseases, which have disastrous health consequences (Teklay, 2015). Despite enormous efforts to control, infectious diseases caused by bacteria, fungi, viruses and parasites continue to pose a serious threat to animal health. Because of a lack of modern medicines and the emergence of widespread drug resistant strains, the impact is particularly severe in developing countries. As a result, animal disease remains one of the leading causes of poor livestock performance (Zampinia et al., 2009).

In Ethiopia, nearly 90% of livestock owners use traditional methods to treat animal diseases. A number of plant species have been identified as having pharmacological activities, and the active ingredients are primarily extracted from different parts of the plant, which are then processed and administered via appropriate routes (Yigezu et al., 2014).

Traditional animal healthcare practices called ethno veterinary medicine provide low cost alternatives in the situation where drug and veterinary services are not available or are too expensive. These practices were developed and practice through trial and error method. However, they have not been evaluated, standardized and documented well. For this reason, appropriate place has not been given in the mainstreaming of veterinary medicine (Dayal et al., 2012). Plants are quite commonly used in traditional practices since medicinal plants represent a rich source of antimicrobial agents. Plants are the source of many potent and powerful drugs. A wide variety of parts have been used for extract as raw drugs, and they all have different medicinal properties. The various parts (root, stem, bark, leaf, seed, flower, fruit and twig exudates) are used. Some of these raw drugs are collected in small quantities and used to treat certain diseases within communities, whereas others are collected in large quantities and traded on the market as raw materials for many herbal industries (Abdallah et al., 2017).

Plants are the basis for medicines by containing natural source of antimicrobial drugs that provide novel or lead compounds for the fight against diseases (Mengiste et al., 2014). These bioactive compounds of plants include alkaloids, flavonoids, tannins and phenolic compounds. Phytochemicals isolated from the medicinal plants show different antimicrobial activities. Because phytochemicals differ in structure from antibiotics, they have different modes of action (Bhatnager et al., 2015).

Ferula is a food plant that is also used in traditional medicine to treat animal diseases. *Ferula communis L*., also known as giant fennel, has long been used to treat a variety of ailments in traditional medicine. Fresh plant materials, crude extracts and isolated components of *F. communis* are used as drugs (Gamal & Atraiki, 2015). Several authors studied *F. communis'* botanical properties, photochemistry, pharmacology and toxicology to determine its therapeutic and toxic potential. The phytochemical component and antimicrobial effect of the plant may vary depending on the soil and climate in which it grows (Mamoci et al., 2011). There was no attempt to determine phytochemicals and antibacterial activity of the plant in Northwest part of Ethiopia.

## 2 MATERIALS AND METHODS

### 2.1 Plant collection, identification and characterization

The plant was collected in the Gondar Zuria district of north‐western Ethiopia. An experienced botanist identified and characterized the plant. This study made by using of the plant's root bark.

### 2.2 Plant preparation for extraction and maceration

After the plant was collected, the root bark was used for work. To remove the dirt and soil, it was washed with water. The root was pulverized and stored in a shed. It was then cut into small pieces with a knife and a scalpel blade. In vitro antibacterial activities of 80% methanol crude extracts were prepared from the root bark of *F. communis* (Nn, 2015). In this study, we socked plant material with 80% methanol and 80% ethanol for three days. The mixture was then filtered through double layer gauze into another container. To obtain a clear extract, the filtration process was repeated three times. Finally, it was kept at +4°C until it was used.

### 2.3 Preliminary phytochemical screening

#### 2.3.1 Test for tannins

A small amount of the extract (0.25 g) was heated in a water bath with 10 mL of distilled water. Three drops of 0.1% ferric chloride were added to the filtrate after the mixture was filtered. The presence of tannins in distilled water was indicated by a blue, blue black, green or blue green solution or precipitates (Ayoola1 et al., 2008).

#### 2.3.2 Test for saponins

A 0.2 g plant extract of *F. communis* was mixed with 5 mL distilled water and vigorously shacked. Saponins were detected by stable persistent frothing (the appearance of a creamy mix of small bubbles) (Ayoola1 et al., 2008).

#### 2.3.3 Test for terpenoid

The *F. communis* plant extract (0.2 g) was mixed with 2 mL of chloroform, and 3 mL of concentrated H_2_SO_4_ was carefully added to form a layer. A reddish‐brown interface was formed which indicated the presence of terpenoid on both extract. Test for flavonoids: About 0.2 g of the extract was dissolved with ferric chloride. Formation of blackish red colour indicated the presence of flavonoids (Ahuja et al., 2013)

#### 2.3.4 Test for steroid

Acetic anhydride (2 mL) was added to 0.5 g of the *F. communis* plant extract in a test tube. It was then followed by the addition of 2 mL of sulphuric acid. A colour change from violet to blue or green indicated the presence of steroids on both extract.

#### 2.3.5 Test for coumarin

One gram of extract was kept with water. Then it was divided into two test tubes. One with 10% ammonium hydroxide added and another as a control. If fluorescence colour indicted positive for coumarin (Ahuja et al., 2013).

#### 2.3.6 Test for cardiac glycosides

A small amount of the extract was hydrolysed in 2 mL of HCl solution and neutralized with equal amount of Sodium hydroxide solution. Few drops of Fehling's solution A and B were added. Red precipitates indicated the presence of glycosides.

#### 2.3.7 Test for anthraquinone

About 0.5 g *F. communis* of sample of each plant extract was shaken with 5 mL of chloroform and filtered. A 10% ammonium hydroxide solution (5 mL) was added to the filtrate, and the mixture was shaken. The presence of a pink, red, or violet colour was taken as an indication of the presence of anthraquinone (Aiyelaagbe & Osamudiamen, 2009).

#### 2.3.8 Test organisms

Standard organisms obtained from American Type Culture Collection or reference strains were used. *Staphylococcus aureus*, *Citrobacter*, *Pseudomonas aeruginosa*, *Salmonella typhi* and *Klebsiella pneumoniae* were standard bacterial species used in this study but *Escherichia coli* was clinically collected bacteria from calf diarrhoea.

### 2.4 Antibacterial assay

The initial screening of antimicrobial activity and determination of minimum inhibitory concentration (MIC) for different extracts were performed by Mueller Hinton agar plate diffusion method and macro‐broth dilution method, respectively (Shubha & Hiremath, 2010). The organisms spread by pour plate method using sterile cotton swab. In each plate wells of 6 mm diameter were made using a sterile borer. Bacterial concentration of 1 × 10^8^ CFU/mL was used for antibacterial activity. The extracts were freshly reconstituted with dimethyl sulphide. The wells were filled with 50 μL of diluted extracts at 500 mg/mL with four different concentrations by serial dilution of 250, 125 and 62.5 mg/mL. Take 50 μL plant extract with micro pipette from each concentration and added. Antibacterial assay plates were incubated at 37 ± 1°C for 24 h. Dimethyl sulphide was used as negative control and standard antibiotics chloramphenicol and ampicillin (0.1 mg/mL) used as positive control. Diameter of the zone of inhibition (in mm) surrounding each well was recorded (Patil et al., 2015).

### 2.5 Determination of minimum inhibitory concentration

The MICs of plant extracts were determined using the Mueller Hinton Broth micro‐dilution method in 96‐well microtitre plates. Micro culture tetrazolium assay reagent has been used effectively to differentiate between live and dead bacteria because only live bacteria convert the dye into an insoluble purple formazan measured at 560 nm. One ml of broth was mixed with 100 μL of bacterial suspension, of which 50 μL was used during transferred to 96 well microtitre plat for one plate. First 50 μL broth added to the plat up to 12 well then 50 μL plant extract was added with it up to 11 well then it was made a dilution supplemented by serial doubling dilutions of the extract. Finally, 50 μL of bacterial suspension (108 CFU/mL) was mixed up to 10 well. The plates were wrapped loosely with cling film to ensure that the bacteria did not get dehydrated and then they were placed in an incubator at 37°C for 24 h. After 24 h of incubation, the plats were removed from incubator and adding of tetrazolium chloride by preparing as liquid and again incubated and was kept for 30 min then observe the colour change visually or subculture with plate count agar and observe growth.

### 2.6 Minimum bactericidal concentration

The minimum bactericidal concentration (MBC) corresponded to the lowest concentration that yields negative subcultures after incubation at appropriate temperature of 37°C for 24 h. This was taken from last growth in MIC subculture with plate counter agar (PCA), that means determined in broth dilution tests by subculturing 10 μL from negative wells cultured on PCA medium. Dividing the medium into four parts from the Petridis opposite side with marker (Bouyahya et al., 2016).

### 2.7 Data analysis

The experimental data are expressed in mean ± Standard Error of the Mean. Data were analysed using Statistical Package for the Social Sciences (SPSS), version 22.0 software. The statistical differences of the mean zone of inhibition of crude extract and solvent fractions for individual bacterium were carried out by one‐way analysis of variance followed by Tukey post hoc multiple comparison test at a significance level of *p* < 0.05.

## 3 RESULTS

### 3.1 Preliminary phytochemical screening

The result of preliminary phytochemical screening test is shown in Table 1. According to the qualitative phytochemical screening test, ethanol extracts were positive for flavonoids, tannins and coumarins. The methanol extract was positive for the presence of tannins, flavonoids, coumarins and terpenoids. The ethanol extract of *F communis* highly positive for tannin.

**TABLE 1:** Preliminary phytochemical screening results of methanol and ethanol extracts.

Metabolites tested	Ethanol extract	Methanol extract
Flavonoids	+	+
Tannins	++	+
Coumarins	+	+
Terpenoids	−	+
Saponins	−	−
Cardiac glycosides	−	−
Steroids	−	−
Anthraquinones	−	+

*Note*: ‘+’ indicates present, ‘−’ indicates absence and ‘++’ indicates availability of phytochemical in large amount.

### 3.2 Agar diffusion test

Both methanol and ethanol extract inhibited the growth of all tested bacterial species. The inhibition was measured in a concentration‐dependent manner. Inhibition was achieved at plant concentrations of up to 125 mg/mL. *Salmonella typhus* was more susceptible than any other gram‐negative bacteria in methanol extract, with a concentration of 500 mg/mL, followed by *E. coli*, *Klebsiella pneumonia* and *Pseudomonas erogenous*. *Citrobacter* had a minimum zone of inhibition of 2.67 mm, whereas ethanol extract of *F. communis* had a slightly similar antimicrobial effect on gram‐negative bacteria except *P. aeruginosa*, with a minimum zone of inhibition of around 2.67 mm. Gram‐positive bacteria, *S. aureus*, were also inhibited slightly better by methanol extract than by ethanol extract. The inhibition zones in methanol were 11 and 10.7 mm in ethanol extract. In general, the *F. communis* plant extract in both fractions was more effective against gram positive bacteria than gram‐negative bacteria. The antibacterial activity pattern of the methanol extract on *S. aureus* was significant (*p* < 0.05) at 500 mg/mL within a group. The antimicrobial effect in ethanol extract was similar with methanol extract both in gram negative and gram positive except slightly variation within concentration difference and ethanol extract showed low inhibition on *P. aeruginosa*. Except for *K. pneumonia* and *S. aureus*, there was no difference in inhibition zone between positive control and 500 mg/mL concentration (Tables 2 and 3).

**TABLE 2:** Zone of inhibition of the different concentrations of methanol extracts.

Concentration	*Citrobacter*	*Salmonella typhi*	*Pseudomonas aeruginosa*	*Escherichia coli*	*Klebsiella pneumonia*	*Staphylococcus aureus*
500 mg/mL	6 ± 3.0	10.33 ± 0.7^dc^	8.33 ± 0.	8.67 ± 0.3^cd^	8.33 ± 0.3^fdcb^	11 ± 1.0^fd^
250 mg/mL	5.3 ± 2.7	8 ± 0.6^fdc^	2.33 ± 2.3^fa^	5.67 ± 2.8^f^	5.33 ± 1.3^fdca^	9.33 ± 1.2^fd^
125 mg/mL	2.3 ± 2.3	2.33 ± 2.3^afb^	0.0 ± 0.0^fa^	0.00 ± 0.0^fa^	0.0 ± 0.0^fba^	6 ± 3.0^f^
62.5 mg/mL	1.3 ± 1.3^f^	0.0 ± 0.0^fba^	0.0 ± 0.0^fa^	0.0 ± 0.0^dfa^	0.00 ± 0.0^fba^	0.0 ± 0.0^fab^
Ampicillin	11.3 ± 0.3^d^	13.3 ± 0.3^bcd^	12 ± 1.1^bcd^	–	–	–
Chloramphenicol	–	–	–	13.67 ± 0.9^dcb^	13.3 ± 1.3^dcba^	21.3 ± 1.3^dcab^

*Note*: Where ‘a’ compared 500 mg/mL, ‘b’ 250 mg/mL, ‘c’ 125 mg/mL, ‘d’ crude 62.5 mg/mL, ‘f’ to positive control and *p* < 0.05, The negative control has shown no antibacterial activity, standard (ATCC) strains.

**TABLE 3:** Zone of inhibition (mm) of the different concentrations of ethanol extracts.

Concentration	*Citrobacter*	*Salmonella typhi*	*Pseudomonas aeruginosa*	*Escherichia coli*	*Klebsiella pneumonia*	*Staphylococcus aureus*
500 mg/mL	6 ± 3.0	10.33 ± 0.7^dc^	8.33 ± 0.	8.67 ± 0.3^cd^	8.33 ± 0.3^fdcb^	11 ± 1.0^fd^
250 mg/mL	5.3 ± 2.7	8 ± 0.6^fdc^	2.33 ± 2.3^fa^	5.67 ± 2.8^f^	5.33 ± 1.3^fdca^	9.33 ± 1.2^fd^
125 mg/mL	2.3 ± 2.3	2.33 ± 2.3^afb^	0.0 ± 0.0^fa^	0.00 ± 0.0^fa^	0.0 ± 0.0^fba^	6 ± 3.0^f^
62.5 mg/mL	1.3 ± 1.3^f^	0.0 ± 0.0^fba^	0.0 ± 0.0^fa^	0.0 ± 0.0^dfa^	0.00 ± 0.0^fba^	0.0 ± 0.0^fab^
Ampicillin	11.3 ± 0.3^d^	13.3 ± 0.3^bcd^	12 ± 1.1^bcd^	–	–	–
Chloramphenicol	–	–	–	13.67 ± 0.9^dcb^	13.3 ± 1.3^dcba^	21.3 ± 1.3^dcab^

*Note*: Where ‘a'compared 500 mg/mL, ‘b’ to 250 mg/mL, *p* ≤ 125 mg/mL, ‘d’ to crude 62.5 mg/mL, ‘f’ to positive control and *p* < 0.05. The negative control has shown no antibacterial activity, standard (ATCC) strains.

### 3.3 Minimum inhibitory concentration and minimum bactericidal concentration

**FIGURE 1:**
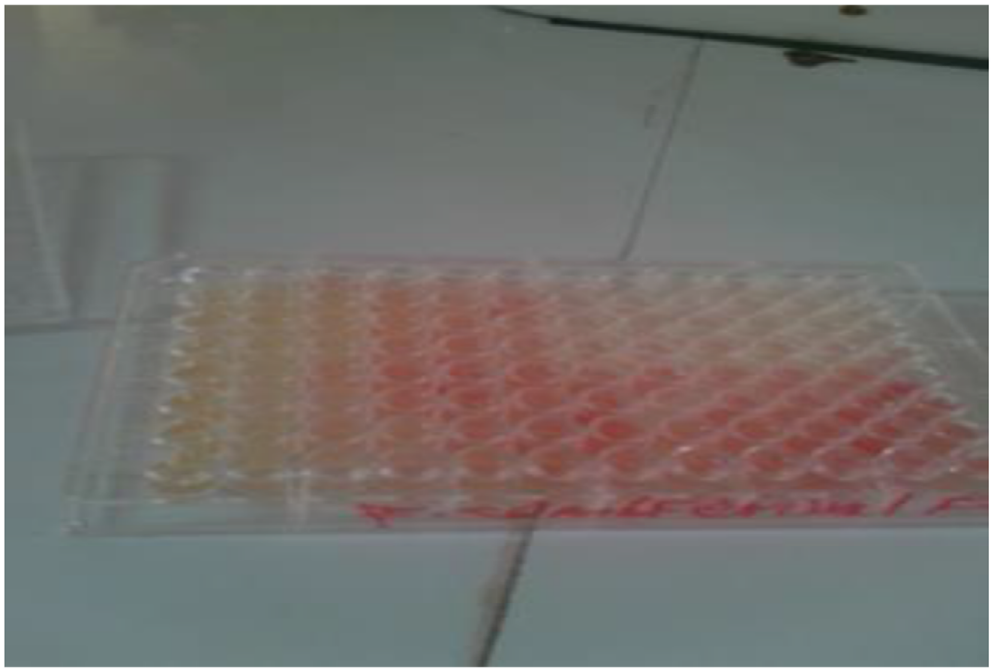
Photo taken from minimum bactericidal concentrations of ethanol extract within 96 well plates.

**FIGURE 2:**
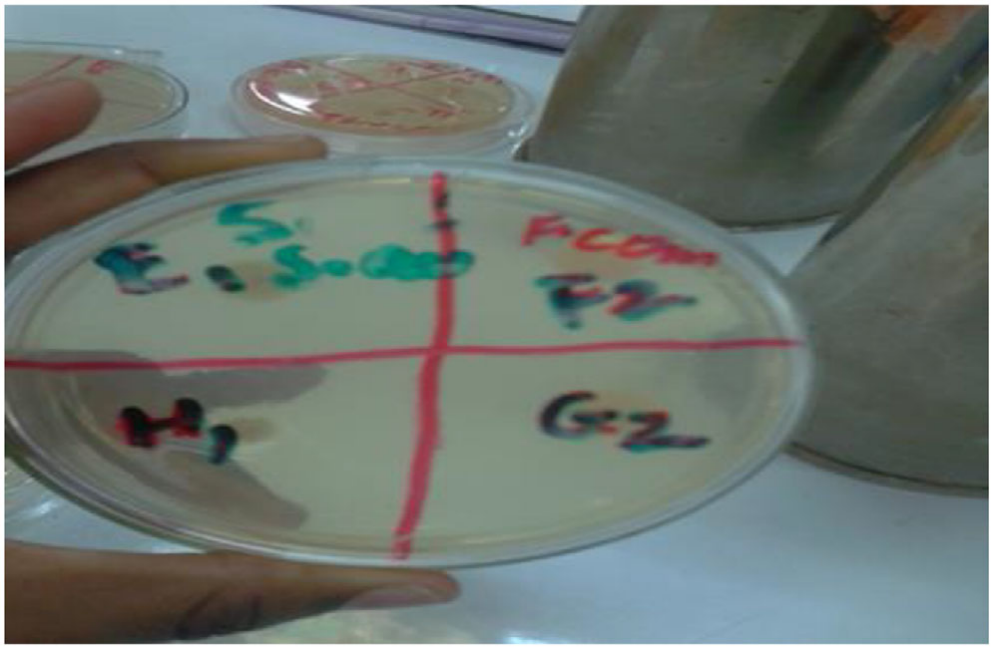
Photo taken from minimum inhibitory concentrations of ethanol extract in plate count agar.

The MIC and MBC of methanol extract were lower in concentration than ethanol extract except *S. aureus* and *Citrobacter*. The mean MIC and MBC (in mg/mL) has almost similar amount (Figures 1 and 2). Table 4 provides a detailed explanation and results.

**TABLE 4:** The MIC and MBC (in mg/mL) of the methanol and ethanol extract.

Tested bacterial strain	Plant extract			
	Methanol		Ethanol	
	MIC	MBC	MIC	MBC
*Escherichia coli*	47.33 ± 1.76	47.33 ± 1.76	48.67 ± 2.44	48.67 ± 2.44
*Salmonella typhi*	32.33 ± 2.84	32.33 ± 2.84	33.3 ± 2.66	33.3 ± 2.66
*Staphylococcus aureus*	18.66 ± 1.33	18.66 ± 1.33	16.67 ± 1.76	16.67 ± 1.76
*Klebsiella pneumoniae*	42.67 ± 3.25	42.67 ± 3.25	44 ± 2.33	44 ± 2.33
*Citrobacter*	78.67 ± 2.33	78.67 ± 2.33	76 ± 3.5	76 ± 3.5
*Pseudomonas erogenous*	50.67 ± 3.25	50.67 ± 3.25	70 ± 1.15	70 ± 1.15

Abbreviations: ATCC, American type culture colony; MBC, minimum bactericidal concentration; MIC, minimum inhibitory concentration.

## 4 DISCUSSION

Plants have been found to contain over 2000 phytochemicals. The medicinal value of plants is determined by the chemicals found in them (Nn, 2015). *F. communis* is a medicinal plant that is used to treat a variety of diseases. In this study, the methanol and ethanol extracts of *F. communis* root bark were positive for flavonoid, tannin and coumarin in this study, whereas the methanol solvent fraction was positive for tannins, flavonoids, coumarin, terpenoid and anthraquinone but negative for saponins, steroid and cardiac glycosides. According to other reports, the phytochemical analysis of *F. communis* Ethyl acetate extract and *n*‐butanol extract revealed the presence of flavonoids, alkaloids, terpenoid, diterpenes, glycosides, terpenoid, phlobatannins and tannins (Gamal & Atraiki, 2015; Maggi et al., 2009). The presence of different chemicals may be due to differences in the type of solvent, extraction method, soil and age of the plant. According to (Hu et al., 2017), the presence of coumarin, terpenoid, essential oils and highly lipophilic compounds in plants causes, antibacterial activity to be more effective (Kang et al., 2012; Waksmundzka‐hajnos et al., 2012).

The present study was undertaken to determine on which extract do the constituents of the root bark of *F. communis* responsible for its antibacterial activity. The antibacterial activities of *F. communis* plant extracts had inhibitory effect against *S. aureus* and gram‐negative bacteria. According to (Akaberi et al., 2015; Gamal & Atraiki, 2015) the aerial part extract of *F. communis* had antibacterial and cytotoxic activities. The *n*‐butanol and ethyl acetate root bark of extract exhibited more interesting antimicrobial activities. This difference may be due to geographical areas of plant collection, extraction method and the parts of the plant used for extraction. According to (Mahendra & Bisht, 2012), a hot water extract of the dried root of *F. communis* in Egypt with a concentration of 200–300 mg/mL had an antibacterial effect similar to the current study with a concentration of 250–500 mg/mL, but there is a minor difference. This variation may be due to geographical areas of plant collection, extraction method and plant parts used for extraction.

The antibacterial activities of *F. communis* plant extracts had positive effect against *S. aureus* and gram‐negative bacteria with a concentration of about 125 mg/mL. At a concentration of 62.5 mg/mL, both gram‐negative and gram‐positive bacteria had a very rare effect. *P. aeruginosa* was more susceptible in methanol extract than ethanol extract and had a lower effective than other gram‐negative bacteria in the current study. This could be due to the extract's mechanism of action on bacteria. In this study, *F. communis* was more potent against gram positive bacteria than that of gram‐negative bacteria.

MIC was 16.67 mg/mL in ethanol extract and 18.66 mg/mL in methanol extract of minimum inhibition in *S. aureus* but in gram‐negative bacteria average MIC above 30 mg/mL. In other study better antibacterial effect of 12 mg/mL and antimycobacterial effect of 8 mg/mL with ethyl acetate and *n*‐butanol extract (Gamal & Atraiki, 2015). The difference may be due to extraction solvent variation and bacterial strain difference. The ethyl acetate and *n*‐butanol extracts of *Ferula asafoetida* had substantial antibacterial activity that is MIC and MBC against *S. aureus* was 1.25 mg/mL (Shubha & Hiremath, 2010) which shows more antimicrobial activities than *F. communis*. The difference may be due to specious of plant, solvent extraction or type of soil.

## 5 CONCLUSIONS AND RECOMMENDATIONS

Medicinal plant species are used to treat diseases of infectious origin. Phytochemicals are chemical compounds produced by plants. So it can be considered a factory of many chemical products. The genus Ferula is one of the plants used in traditional foods as well as in traditional medicine. In this study, *F. communis* plant, which offer a scientific basis for traditional use of medicine in both ethanol and methanol extracts for in vitro antimicrobial effect. The test plants showed antibacterial activity as evidenced by moderate zone of inhibition on both gram‐negative and gram‐positive bacteria except *Citrobacter*. This finding gives hints that potential lead molecule can be isolated from this medicinal plant that can be a base for synthesis of effective antibacterial drugs. Phytochemical screening of the *F. communis* showed the presence of secondary metabolites that are responsible for their antibacterial activity and effectiveness. Based on the findings, further research on plant fractions and isolates for antibacterial activity and toxicities should be made. The plant *F. communis* has many advantages, so it has to be cultivated well and preserved. Awareness creation among the community regarding this plant has paramount importance and should be practice well. Study should be done to understand mode of action of the phytochemicals found in this plant against bacteria and other microbes.

## AUTHORS CONTRIBUTION

In this research paper, Dr. Betelihem Yirdaw participated in conceptualization, data collection, laboratory work and manuscript write‐up, whereas Dr. Temesgen Kassa contribute to data analysis, edition and validation.

## CONFLICT OF INTEREST STATEMENT

No conflict of interest between the authors

## FUNDING INFORMATION

This research did not receive a specific fund

### PEER REVIEW

The peer review history for this article is available at https://publons.com/publon/10.1002/vms3.1170.

## Data Availability

Data will be available when necessary
